# Intensive care–treated cardiac arrest: a retrospective study on the impact of extended age on mortality, neurological outcome, received treatments and healthcare-associated costs

**DOI:** 10.1186/s13049-021-00923-0

**Published:** 2021-07-28

**Authors:** Ester Holmström, Ilmar Efendijev, Rahul Raj, Pirkka T. Pekkarinen, Erik Litonius, Markus B. Skrifvars

**Affiliations:** 1 Department of Emergency Care and Services, University of Helsinki and Helsinki University Hospital, Helsinki, Finland; 2 Department of Anaesthesiology, Intensive Care and Pain Medicine, University of Helsinki, Helsinki University Hospital, Helsinki, Finland; 3 Department of Neurosurgery, University of Helsinki and Helsinki University Hospital, Helsinki, Finland

**Keywords:** Elderly, Cardiac arrest, intensive care unit, critical care, cardiopulmonary resuscitation, OHCA, IHCA, ICUCA

## Abstract

**Background:**

Cardiac arrest (CA) is a leading cause of death worldwide. As population ages, the need for research focusing on CA in elderly increases. This study investigated treatment intensity, 12-month neurological outcome, mortality and healthcare-associated costs for patients aged over 75 years treated for CA in an intensive care unit (ICU) of a tertiary hospital.

**Methods:**

This single-centre retrospective study included adult CA patients treated in a Finnish tertiary hospital’s ICU between 2005 and 2013. We stratified the study population into two age groups: <75 and $\ge$75 years. We compared interventions defined by the median daily therapeutic scoring system (TISS-76) between the age groups to find differences in treatment intensity. We calculated cost-effectiveness by dividing the total one-year healthcare-associated costs of all patients by the number of survivors with a favourable neurological outcome. Favourable outcome was defined as a cerebral performance category (CPC) of 1–2 at 12 months after cardiac arrest. Logistic regression analysis was used to identify independent associations between age group, mortality and neurological outcome.

**Results:**

This study included a total of 1,285 patients, of which 212 (16 %) were $\ge$75 years of age. Treatment intensity was lower for the elderly compared to the younger group, with median TISS scores of 116 and 147, respectively (*p* < 0.001). The effective cost in euros for patients with a good one-year neurological outcome was €168,000 for the elderly and €120,000 for the younger group. At 12 months after CA 24 % of the patients in the elderly group and 47 % of the patients in the younger group had a CPC of 1–2 (*p* < 0.001). Age was an independent predictor of mortality (multivariate OR = 2.90, 95 % CI: 1.94–4.31, *p* < 0.001) and neurological outcome (multivariate OR = 3.15, 95 % CI: 2.04–4.86, *p* < 0.001).

**Conclusions:**

The elderly ICU-treated CA patients in this study had worse neurological outcomes, higher mortality and lower cost-effectiveness than younger patients. Elderly received less intense treatment. Further efforts are needed to recognize the tools for assessing which elderly patients benefit from a more aggressive treatment approach in order to improve the cost-effectiveness of post-CA management.

**Supplementary Information:**

The online version contains supplementary material available at 10.1186/s13049-021-00923-0.

## Background

CA is one of the leading causes of death in the developed world [1], with over three million patients affected each year worldwide [2]. This, in addition to a clear increase in patient longevity globally, mandates more research efforts towards care of the elderly post-CA [3, 4]. Not unexpectedly, cardiopulmonary resuscitation (CPR) is more commonly initiated in younger patients, and younger patients receive more aggressive treatment by mobile medical teams [4, 5]. Although post-CA mortality increases with age, it has been disputed whether this is due to age in itself or other CA characteristics [6–8]. Pre-arrest comorbidity and CA factors still need more research in order to explain the variability of outcome in CA among elderly [9].

There is limited data published on the actual costs related to CA including care in the hospital as well as rehabilitation [10]. Increasing longevity in combination with decreasing mortality leads to increasing healthcare costs, thus putting a burden on health care systems [11, 12]. Very few studies have focused on the post-CA treatment of the elderly in the intensive care unit (ICU) while including long-term outcome [3, 13]. A comprehensive assessment of total costs is a first step enabling a more cost-effective use of resources. Accordingly, we designed the current study to explore treatment intensity, outcome and healthcare-associated costs of the ICU-treated elderly CA patients treated in a single centre over a 9-year period. We hypothesised that compared with younger patients, the elderly have higher mortality and worse neurological outcome despite high treatment costs and intensity.

## Methods

### Study design and setting

This retrospective cohort study was conducted at Meilahti Hospital, Helsinki, Finland, which serves as the primary referral centre for CA patients in the Helsinki and Uusimaa region. This region has a population of approximately 1.7 million people (30 % of the total Finnish population). Data were extracted from the Finnish Intensive Care Consortium (FICC) database [14] and include adult CA patients ($\ge$18 years of age) treated in the hospital’s ICU between January 1, 2005 and December 31, 2013. We reviewed Electronic health records (EHR) of individual patients for relevant data. Patients with incomplete or missing data and patients where return of spontaneous circulation (ROSC) was not achieved were excluded from the analyses. The patients were divided into two age groups for descriptive purposes: <75 (young) and $\ge$75 years (elderly).

 The study was approved by the ethics committee of the Operative Division of Helsinki University Hospital (June 2014: 194/13/03/02/2014 TMK02 § 97), the Finnish National Institute for Health and Welfare (May 2014: THL/713/5.05.01/2014), Statistics Finland (May 2014: TK-53-1047-14), the Social Insurance Institution (September 2015: Kela 55/522/2015) and the Office of the Data Protection Ombudsman (February 2016: 2794/204/2015).

### Data collection and extracted variables

The FICC database provided data on hospital survival, preadmission physical status (a modified World Health Organization/Eastern Cooperative Oncology Group (WHO/ECOG) classification implemented by FICC), mean TISS-76 score and its components for the complete ICU stay, and Acute Physiology and Chronic Health Evaluation II (APACHE II) components and scores [15–18]. In this study APACHE II scores were used excluding the points for age; thus including points for body temperature, mean arterial pressure, pH, heart rate, respiratory rate, sodium, potassium, creatinine, acute renal failure, hematocrit, white blood cell count, Glasgow coma scale and fraction of inspired oxygen within 24 h of admission to the ICU [16]. We obtained the confirmed date of death by linking the patients’ unique personal identification numbers with the Finnish Population Register Centre database, which registers all deaths of Finnish residents. Detailed information regarding preadmission physical status, time of CA, time to ROSC, initial CA rhythm and location was collected from the hospital’s EHR. The Cerebral Performance Category (CPC) score for survivors at one year after CA was assessed using these same EHRs as most patients had sought medical attention for other reasons 12 months post-CA, and thereby had health records recording their rough neurological status [19–22]. We determined preadmission functional status by using a simplified WHO/ECOG classification, where “independent” was defined as the patient being independent in self-care and “dependent” was defined as the patient being partly or fully dependent on help in self-care prior to hospital admission [23]. A favourable neurological outcome was defined as CPC scores of 1–2 and an unfavourable neurological outcome as CPC scores of 3–4 [22].

### Healthcare-associated costs

Healthcare-associated costs included three parameters: index hospital costs, rehabilitation costs and social security costs. We obtained hospital costs from the hospital’s billing records. These included costs incurred during the entire treatment period, such as costs of personnel, surgery, diagnostics as well as ICU and ward stay. Rehabilitation costs were calculated by multiplying the length of stay (LOS) in the rehabilitation unit with the average cost per day for the respective level of care unit [24]. Social security costs were retrieved from the national Social Insurance Institution. This is a government-based social security and healthcare system. All reimbursements made by the Social Insurance Institution, up to one year after the admission, were obtained and summed. These included disability allowances, sickness allowances, private physician and physiotherapist expenses, prescription drug expenses and medical transport expenses. All costs were converted to euros based on the 2021 currency rate in order for costs to be more easily interpreted and comparable with more recent patient data and research. Cost data analysis included the calculation of median healthcare costs for each age group and separately for the survivors with a favourable neurological outcome in the studied age groups.

Effective cost per survivor with favourable neurological outcome (ECPSFNO) was calculated by dividing the sum of the total cost for all patients within each age group by the number of patients within that group with a favourable neurological outcome (CPC of 1–2) after 12 months [25]. We further stratified costs according to the location of CA (out-of-hospital CA (OHCA), in-hospital CA (IHCA) and in-ICU CA (ICUCA)). A mean of total, hospital, rehabilitation and social insurance institution costs was separately calculated for each age group and illustrated using bar-charts.

### Statistical analysis

For statistical analyses we used SPSS statistics for MAC, version 25.0, released in 2017 (IBM Corp, Armonk, NY, USA). The baseline characteristics of the study cohort are described using proportions with percentiles for categorical values and medians with interquartile range for continuous variables. We tested group differences with Mann-Whitney U-test or Chi-square test, as appropriate. Logistic regression was used to calculate univariable odds ratios with corresponding 95 % confidence intervals regarding impact on mortality, neurological outcome and costs. A p-value under 0.05 was defined as significant. Significant factors were included in a multivariate regression model to identify independent predictors of unfavourable neurological outcome and mortality. We illustrated the difference in mortality between the two age groups by using Kaplan Meyer survival curves and a clustered bar of cumulative percentages.

Chi-square tests were used on TISS-point distribution to determine if there were significant differences in treatment intensities between the two age groups and if the location of CA (OHCA or IHCA) affected/influenced treatment intensity. We performed a multivariate regression model in order to find independent factor’s impact on total-, hospital-, rehabilitation- and Social Insurance Institution costs.

## Results

### Study population and factors at resuscitation

The study included 1,285 patients, of which 212 (16 %) were 75 years or older and 1,073 (84 %) younger than 75 years (Table 1). OHCAs were less common among the elderly with an occurrence of 43 % compared to 64 % in the young group, *p* < 0.001 (Table 1). A number of other differences between the elderly and the younger population were noted: fewer elderly patients had an independent preadmission functional status (75 % vs. 90 %, *p* < 0.001), a non-shockable initial CA rhythm was more common (49 % vs. 35 %, *p* < 0.001), and ROSC was achieved faster among the elderly patients (median of 10 min vs. 16 min, *p* < 0.001).

**Table 1:** Patients characteristics

	Age < 75 (n = 1073)	Age ≥75 (n = 212)	*p*
Women, % (n)	26 (281)	33 (70)	0.041
Location of arrest, % (n)			< 0.001
OHCA	64 (691)	43 (92)	
IHCA	27 (286)	46 (97)	
ICUCA	9 (96)	11 (23)	
Witnessed arrest, % (n) ^a^	87 (935)	91 (193)	0.130
Initial cardiac-arrest rhythm, % (n)			< 0.001
Shockable (VT or VF)	60 (641)	44 (94)	
Non-Shockable (all other rhythms)	35 (378)	49 (104)	
Unknown	5 (54)	6.6 (14)	
Time to ROSC in minutes, median (IQR) ^b^	16 (10–23)	10 (5–18)	< 0.001
Independent preadmission functional status % (n)^c^	90 (960)	75 (158)	< 0.001

^a^ 2 % of patients are missing this information

^b^ 9,5 % of patients are missing this information

^c^ 4.7 % of patients are missing this information

### Treatment intensity & ICU factors

No difference was observed in the APACHE II scores between the elderly and younger patients when points for age where excluded (Table 2). Treatment intensity was lower in the elderly than in the younger age group, with median daily average TISS scores of 34 and 37 for the elderly and younger patients, respectively, *p* < 0.001. The total amount of TISS points was also lower for the elderly (116 vs. 147, *p* < 0.001) (Table 2). In-hospital as well as in-ICU mortality was higher for the elderly group (ICU mortality 33 % vs. 18 %, *p* < 0.001; hospital mortality 49 % vs. 33 %, *p* < 0.001). The ICU LOS was shorter for the elderly than for the younger patients (Table 2). The ICU LOS among the survivors was however not different. Table 2 details the ICU factors, in-hospital mortality, TISS-point distribution and the difference in the selected treatments received at the hospital. TISS-point distribution can be viewed in more detail in the supplementary material (Additional file 1, 2 and 3).

**Table 2:** Intensive care unit-factors

	Age < 75 (n = 1073)	Age ≥75 (n = 212)	*p*
APACHE II-score excluding age points, median (IQR)	20 (15–27)	22 (15–27)	0.181
TISS-Score, median (IQR)			
Daily average	37 (31–43)	34 (28–41)	< 0.001
Total TISS-score	147 (93–227)	116 (65–192)	< 0.001
Treatments received, % (n)			
Controlled ventilation with or without positive end-expiratory pressure	98 (1055)	93 (197)	< 0.001
Induced hypothermia	42 (450)	16 (34)	< 0.001
Vasoactive drug infusion (> 1 drug)	47 (503)	38 (80)	0.015
Continous antiarrhythmia infusions	20 (212)	17 (35)	0.273
Seizure treatment	13 (140)	6 (13)	0.004
Hemodialysis in unstable patient	2 (18)	4 (8)	0.048
Arterial line	100 (1071)	100 (211)	0.432
In-hospital mortality %(n)			
Dead in ICU	18 (194)	33 (69)	< 0.001
Dead in hospital	33 (357)	49(104)	< 0.001
Length of stay in days, median (IQR)			
ICU	3 (2–5)	2 (1–4)	< 0.001
Hospital	10 (4–20)	8 (3–16)	0.003
Length of stay in days among patients discharged alive, median (IQR)			
ICU	3 (2–6)	3 (2–6)	0.085
Hospital	14 (8–24)	14 (8–23)	0.654

### Healthcare-associated costs

**Fig. 1:**
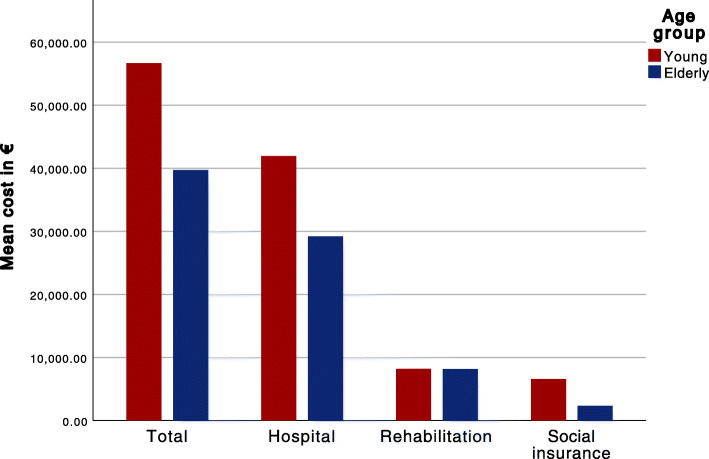
Mean cost distribution among all patients in 2021 euros

The ECPSFNO was €168,000 and €120,000 for the elderly and young group, respectively. The effective cost for the elderly patient group was higher than that for the younger patient group in all locations of CA except for ICUCA, where it was €173,000 and €308,000, respectively. The elderly patient group received less median funding from the Social Insurance Institution, €714 compared to €1,670 in the younger age group (Table 3; Fig. 1, Additional file 4). Median rehabilitation costs were higher for the elderly patient group when we only included those with a favourable 12-month neurological outcome, €6,070 compared to €2,110 (*p* = 0.012) (Table 3). An additional table of the independent predictors of total-, rehabilitation-, hospital- and Social Insurance Institution funding can be viewed in the supplementary material (Additional file 5).

**Table 3:** Resource use

	Age < 75 (n = 1073)	Age ≥75 (n = 212)	*p*
Cost of treatments in €, median (IQR)			
Hospital costs	29 971 (13 381 − 50 212)	18 356 (9 740 − 37 102)	< 0.001
Rehabilitation	0 (0–6 543)	0 (0–7 574)	0.928
Social Insurance Institution	1 669 (579-6 686)	714 (392-1 760)	< 0.001
Cost of treatment of those with CPC 1–2 after 12 months in €, median (IQR) (57 %)			
Hospital	41 194 (27 031–63 709)	34 888 (19 083 − 60 029)	0.071
Rehabilitation	2113 (0–9 603)	6073 (417 − 13 319)	0.012
Social Insurance Institution	4 561 (1 173 − 15 043)	2 049 (1 196-3 509)	< 0.001
Total cost in €, median (IQR)			
Everyone	38 195 (16 505-71680)	22 641 (12 488 − 47 006)	< 0.001
Those with CPC 1–2 after 12 months (57)	54 510 (36 148 − 86 461)	39 482 (24 101 − 93 020)	0.040
Effective cost^a^ in €			
Of those with CPC 1–2 after 12 months	119 941	168 416	-
Effective cost in € among those with CPC 1–2 after 12 months			
OHCA	90 499	133 134	-
IHCA	161 670	199 540	-
ICUCA	308 000	172 595	-

^a^Effective cost: The total healthcare-associated costs of all patients within their respective age group divided by the number of survivors with a favourable neurological outcome

### Neurological outcome and mortality

**Fig. 2:**
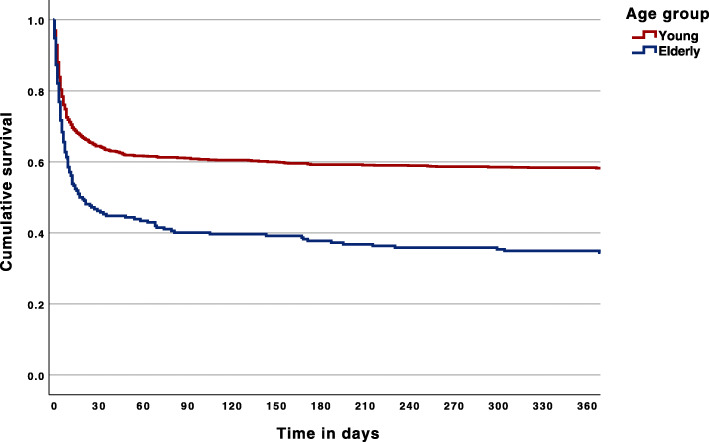
Kaplan-Meier survival curve of all cases during the first year, Log Rank *p* < 0.001

Neurological outcome was worse for the elderly group, with only 24 % (50/212 patients) having CPC scores of 1–2 after 12 months, compared with 47 % (507/1073 patients) of the younger age group, *p* < 0.001. Long-term mortality was higher for the elderly group compared to the younger group; 70 % of the elderly (vs. 44 %) had died within two years, *p* < 0.001. Mortality in the elderly versus the younger age group during the first year is shown in Fig. 2. Separate Kaplan Meier curves illustrating mortality during the whole follow-up period for all patients, patients based on location of arrest (OHCA, IHCA and ICUCA) as well as based on initial rhythm (shockable and non-shockable) can be found in the supplementary material (Additional file 6–7). The median follow-up time was 1.6 years per patient.

Factors independently associated with unfavourable neurological outcome was age above 75 years (OR = 3.15, 95 % CI: 2.04–4.86, *p* < 0.001), dependent pre-admission functional status (OR = 3.00, 95 % CI: 1.62–5.57, *p* < 0.001), non-shockable initial CA rhythm (OR shockable rhythm = 0.43, 95 % CI: 0.31–0.61, *p* < 0.001), location of arrest (OR IHCA = 1.50, 95 % CI: 1.01–2.25, *p* = 0.046 and OR ICUCA = 2.82, 95 % CI: 1.45–5.39, *p* = 0.002), time to ROSC in 10-minute intervals (OR = 1.61, 95 % CI: 1.34–1.94, *p* < 0.001), APACHE II score excluding points for age (OR = 1.97, 95 % CI: 1.61–2.40, *p* < 0.001) and total TISS-points (OR = 0.99, 95 % CI: 0.98-1.00, *p* = 0.11).

Factors independently associated with mortality were age above 75 years (OR = 2.90, 95 % CI: 1.94–4.31, *p* < 0.001), dependent pre-admission functional status (OR = 2.37, 95 % CI: 1.36–4.14, *p* = 0.002), initial CA rhythm (OR shockable rhythm = 0.46, 95 % CI: 0.33–0.64, *p* < 0.001), location of CA (ICUCA OR = 3.84, 95 % CI: 2.05–7.19, *p* < 0.001), time to ROSC in 10-minute intervals (OR = 1.63, 95 % CI: 1.37–1.93, *p* < 0.001), APACHE II score excluding age (OR = 2.21, 95 % CI: 1.82–2.69, *p* < 0.001) and total TISS-points (OR = 0.98, 95 % CI: 0.98–0.99, *p* = 0.002. Table 4 details the independent predictors of an unfavourable neurological outcome and Table 5 the independent predictors of mortality.

**Table 4:** Univariate models and multivariate models for risk factors predicting 12-month unfavourable cerebral performance status, n = 980^a^

	Univariate model		Multivariate model	
Variable	OR (95 % CI)	*p*	OR (95 % CI)	*p*
Age				
Young (< 75y)	1		1	
Elderly ( > = 75)	3.09 (2.19–4.36)	< 0.001	3.15 (2.04–4.86)	< 0.001
Pre-admission functional status				
Independent	1		1	
Dependent	4.07 (2.48–6.67)	< 0.001	3.00 (1.62–5.57)	< 0.001
Initial CA-rhythm				
Non-shockable	1		1	
Shockable	0.32 (0.25–0.41)	< 0.001	0.43 (0.31–0.61)	< 0.001
Location of arrest				
OHCA	1		1	
IHCA	1.78 (1.37–2.30)	< 0.001	1.50 (1.01–2.25)	0.046
ICUCA	2.24 (1.47–3.41)	< 0.001	2.82(1.45–5.39)	0.002
Witnessed arrest (not witnessed = 1)	0.47 (0.31–0.70)	< 0.001	0.71(0.44–1.16)	0.171
Time to ROSC in 10-minute intervals	1.15 (1.02–1.29)	0.020	1.61 (1.34–1.94)	< 0.001
APACHE II-score excluding age points^b^	2.51 (2.13–2.95)	< 0.001	1.97 (1.61–2.40)	< 0.001
Total TISS-points^b^	0.99 (0.98-1.00)	0.005	0.99 (0.98-1.00)	0.011

^a^ A total of 980 patients were included. 94 patients had missing CPC, 60 patients had missing functional status, 68 patients had missing initial rhythm, 24 patients had missing if the arrest was witnessed, 118 patients had missing time to ROSC, 1 patient had missing APACHE II-score.

^b^ Each step increases the variable by 10

**Table 5:** Univariate models and multivariate models for risk factors predicting 12-month mortality, n = 1055^a^

	Univariate model		Multivariate model	
Variable	OR (95 % CI)	*p*	OR (95 % CI)	*p*
Age				
Young (< 75y)	1		1	
Elderly ( > = 75)	3.44 (2.44–4.84)	< 0.001	2.90 (1.94–4.31)	< 0.001
Pre-admission functional status				
Independent	1		1	
Dependent	4.20 (2.55–6.92)	< 0.001	2.37 (1.36–4.14)	0.002
Initial CA-rhythm				
Non-shockable	1		1	
Shockable	0.33 (0.26–0.42)	< 0.001	0.46 (0.33–0.64)	< 0.001
Location of arrest				
OHCA	1		1	
IHCA	2.07 (1.61–2.67)	< 0.001	1.36 (0.92–1.99)	0.119
ICUCA	2.29 (1.52–3.449	< 0.001	3.84 (2.05–7.19)	< 0.001
Witnessed arrest (not witnessed = 1)	0.59 (0.40–0.86)	< 0.006	0.72 (0.46–1.15)	0.169
Time to ROSC in 10-minute intervals	1.04 (0.93–1.16)	0.520	1.63 (1.37–1.93)	< 0.001
APACHE II-score excluding age points^b^	2.34 (2.01–2.73)	< 0.001	2.21 (1.82–2.69)	< 0.001
Total TISS-points^b^	0.99 (0.98-1.00)	0.001	0.98 (0.98–0.99)	0.002

^a^ A total of 1055 patients were included. 60 patients had missing functional status, 68 patients had missing initial rhythm, 24 patients had missing if the arrest was witnessed, 118 patients had missing time to ROSC, 1 patient had missing APACHE II-score.

^b^ Each step increases the variable by 10

## Discussion

This current study presents a comprehensive estimation of CA-associated costs including hospital costs, rehabilitation costs and social insurance costs at a tertiary university hospital. Elderly patients received less intensive ICU treatment and had shorter ICU LOS. Long-term survival and functional outcome were lower among the elderly and cost effectiveness was lower in most arrest locations except ICUCAs. This suggests a lower cost-effectiveness of the complex ICU care after OHCA and IHCA in the elderly. On the other hand, as the cost-effectiveness of ICUCA was not higher for the elderly it indicates that pre-emptive ICU care and CPR can indeed be cost-effective regardless of patient age. We believe that our results are important with regards to treatment recommendations even though individual care decisions should always be made on a case-by-case basis.

Both TISS-point distribution and the median total cost are lower for the elderly group. This in combination with an even APACHE-score distribution between the age groups (indicating roughly the same comorbidity pre-CA) indicates that age seems to have been a factor affecting treatment intensity. The difference in total TISS-point-distribution can be affected by the LOS, but the LOS does not explain the difference in average daily TISS-points. We speculate that initial treatment intensity was high for both age groups, but that some treatments were stopped earlier or not started at all in the elderly group due to a perceived poor prognosis, thus decreasing the average daily TISS-score. This likely indicates a daily evaluation of patients care in order to avoid futile care especially in the elderly with multiple comorbidities and frailty [26]. Many studies on post-CA therapies, such as targeted temperature management, have excluded elderly patients [27, 28]. A therapy such as TTM has without doubt side effects and in patients with comorbidities the side effects may outweigh the benefits [27, 29]. Recent studies demonstrate that ROSC rates, one-year survival and favourable neurological outcome at one month among elderly CA patients have increased over time with increase in the proportion of advanced in-hospital treatments (i.e. extracorporeal membrane oxygenation, therapeutic hypothermia and/or percutaneous coronary angiogram/intervention) provided [30]. In our study, in-hospital costs of the total provided treatments were lower for the elderly, even when excluding those with a poor one-year neurological outcome. One could argue that the elderly may not benefit from more aggressive treatment, but age in itself should not affect the administered treatments even if it affects mortality, as neurological outcome seems to remain good for survivors [3]. In this study, the ECPSFNO was higher for the elderly group in all locations of CA except ICUCA. Thus, although fewer resources were used by the elderly, the cost per survivor remained higher than the younger age group owing to the high mortality in the elderly group.

Due to marked differences in healthcare funding, direct comparisons of our results with other studies are difficult. Our results also indicate a clear inter-patient variation. Costs in the range of €20,000–40,000 for ICU-treated CA survivors have been determined in previous studies [31, 32]. Nonetheless, several studies and meta-analyses have shown that age negatively affects post-CA mortality [33]. Long-term survival among elderly CA patients is generally lower than that among younger age groups in the case of OHCA [34, 35].

We also looked at the distribution of costs in three separate categories (hospital costs, rehabilitation costs and social insurance costs) among different age groups. Costs were higher for the younger patient group in all categories except rehabilitation. The difference in hospital costs could be attributable to the elderly receiving less aggressive treatment and having to be in a better initial condition in order to survive CA and be taken to the ICU. Less intensive treatment is needed to attain a favourable outcome if the pre-arrest comorbidities are lower, which also decreases hospital costs. The younger age group probably received more funding from the Social Insurance Institution because they got a paid sick-leave from work. Patients over 68 years of age receive pension, which does not alter if the patient is severely ill and therefore isn’t included in these calculations. The higher risk of early post-CA mortality in elderly patients might also have decreased social insurance reimbursements as compared to the younger patients. We noticed that median rehabilitation costs were higher among the elderly when only including those with a favorable 12-month neurological outcome. This could be an indicator for the elderly having more long-time problems post-CA. We can probably not see the same difference in rehabilitation costs when taking into account all patients as the early mortality among elderly decreases the median rehabilitation costs. The effect of less aggressive treatment on the need for rehabilitation among elderly is something further research could focus on.

Additionally, we demonstrated worse long-term outcome in elderly compared to younger patients following care in the ICU after CA. This difference was the most pronounced in OHCAs but was evident in patients with IHCA as well. Interestingly, this study shows that the same percentage of patients in both age groups had a CPC of 3–4 12 months post-CA, but our multivariable model still indicates that age affects neurological outcome. It is debatable how much old age correlates with worse neurological outcome as the high mortality probably affects the statistics. Previous studies also indicate that there isn’t a difference in neurological outcome among elderly compared to younger CA survivors[3, 35]. It is worth noting that age does not always correlate with outcome and is not in itself an adequate prognostic factor, as two elderly persons of the same age can have very different medical conditions [36]. High frailty and a low performance status have been connected with higher ECPSFNO and mortality in previous studies [10, 37–39]. The increased mortality related to age in this study is indeed partially explained by the pre-admission functional status of the elderly patients; thus, this in combination with age seems to better predict both mortality and neurological outcome. Performance status could be a more precise tool when deciding which patients benefit the most from intensive care and more advanced treatment options.

Another possible tool for risk assessment among the elderly seems to be the initial rhythm, as an initially shockable rhythm predicts a better outcome even among the very elderly, where other prognostic factors seem to fail [40]. Supporting the results of previous studies, initial rhythm was one of the factors with the strongest association with outcome among both the younger and elderly patients in this study as well. Interestingly, in our study the elderly had lower incidences of ventricular fibrillation (VF) and ventricular tachycardia (VT) than the younger age group. This could be related to a difference in the aetiology of the arrests or to mechanisms such as faster conversion of VF/VT to asystole owing to the faster depletion of energy in the aged heart. Such an abnormality has been described in mitochondrial metabolism with ageing in the muscle cells [41]. Previous studies have also shown that bradyarrhythmia-related CA patients were generally older than those with tachyarrhythmia-related CA [42]. The significant difference in location of arrest is also something that affects mortality and neurological outcome as CA aetiology differs depending on where the CA occurred [43]. We speculate that elderly having a higher percentage of IHCAs indicates that they are in a worse pre-arrest state and may therefore also have had a higher amount of unfavourable pre-arrest comorbidities, which could increase mortality. Indeed, previous research has shown that comorbidities affect CA-aetiology and initial rhythm in an unfavourable manner [44]. Unfortunately, as we did not have data on CA aetiology, we cannot explore this further. Factors in IHCA that may decrease mortality compared to OHCAs are shorter times to response and more available treatments, but these do probably not affect mortality as much as the pre-arrest comorbidities seem to do. We also discovered that elderly had a shorter time to ROSC, this could in part be due to more elderly suffering from IHCA and ICUCA, where response times are shorter. We may speculate that resuscitation attempts also are terminated and deemed unsuccessful faster with the elderly patients, which shortens the median time to ROSC.

A major strength of this study is its minimal selection bias owing to socioeconomic factors and personal insurance, as this study was conducted in a setting of government-funded healthcare. However, our data on long-term costs are not comprehensive as the elderly in many cases receives a pension, which is not substituted the same way with social insurance funding in case of sick leave. A limitation and factor affecting the outcome of this study is that the studied population only included patients with ROSC who were treated in the ICU; this immediately excludes patients in such a bad initial state that they did not survive until admission to the ICU. We acknowledge that this study is based on data from patients treated between 2005 and 2013. We do not know of any major changes in post resuscitation treatment since 2013, but we cannot be sure to what degree our results are valid today, and with newer patient cohorts. In addition, we do not have data on DNAR-decisions in patients while they were in the ICU. It is likely that treatments were withdrawn more so in the elderly compared to younger patients and there could be a bias regarding local treatment strategies of elderly patients as this is a single-centre study. Finally, the use of CPC instead of other more detailed neurological outcome measures such as the modified Rankin Scale [45].

## Conclusions

Treatment intensity for the elderly is lower as a group, whereas mortality and the risk for a poor neurological outcome is higher, compared to the younger age group. Care of the elderly in the ICU was less cost-effective in case of OHCA and IHCA but not regarding ICUCA. Further studies should focus on the specific tools for identification of elderly patients who can benefit from a more aggressive treatment approach, enabling an improvement in resource allocation and possibly improving the cost-effectiveness of post-CA ICU care.

## Supplementary information

Additional file 1. Table of TISS-point distribution for individual procedures.

Additional file 2. Table of TISS-point distribution for individual procedures (OHCA-cases only).

Additional file 3. Table of TISS-point distribution for individual procedures (IHCA-cases only).

Additional file 4. Mean cost in euro based on initial rhythm (**A**) shockable rhythms (**B**) non-shockable rhythms.

Additional file 5. Multivariate models for risk factors predicting (**A**) total costs (**B**) hospital costs (**C**) rehabilitation costs (**D**) Social Insurance Institution costs.

Additional file 6. KM-curves based on location of arrest (**A**) all cases during the whole follow up-period, Log Rank *p* < 0.001 (**B**) OHCA, Log Rank *p* < 0.001 (**C**) IHCA, Log Rank *p* = 0.003 (**D**) ICUCA, Log Rank *p* = 0.079.

Additional file 7. KM-curves based on initial rhythm (**A**) Shockable rhythm (VF/VT), Log rank *p* < 0.001 (**B**) Non-shockable rhythm, Log Rank *p* = 0.062.

## Data Availability

Legal restrictions prevent us from making the data publicly available, as it is based on patient registers. The data included in this study are obtained from several databases (The Finnish Intensive Care Consortium, Kela, the Finnish National Institute for Health and Welfare, Statistics of Finland and the five university hospitals in Finland). With appropriate research approval, data can be directly requested from the sources.

## Abbreviations

CA: Cardiac arrest
ICU: intensive care unit
TISS: Therapeutic scoring system
CPC: cerebral performance category
CPR: cardiopulmonary resuscitation
FICC: Finnish Intensive Care Consortium
EHR: electronic health records
ROSC: return of spontaneous circulation
WHO/ECOG: World Health Organization/Eastern Cooperative Oncology Group
APACHE: Acute Physiology and Chronic Health Evaluation
LOS: length of stay
ECPSFNO: Effective cost per survivor with favorable neurological outcome
OHCA: out-of-hospital cardiac arrest
IHCA: in-hospital cardiac arrest
ICUCA: in-intensive-care-unit cardiac arrest
