# Supplementary material for: Intensive care–treated cardiac arrest: a retrospective study on the impact of extended age on mortality, neurological outcome, received treatments and healthcare-associated costs
Source: Scand J Trauma Resusc Emerg Med. 2021 Jul 28;29:103. doi: 10.1186/s13049-021-00923-0 (PMC8317381; doi:10.1186/s13049-021-00923-0)
Supplement: Supplementary file 1 — Table of TISS-point distribution for individual procedures. [file 13049_2021_923_MOESM1_ESM.docx]

**Additional file 1.** Table of TISS-point distribution for individual procedures

| Procedure, % (n) | Age <75 (n =1073) | Age ≥75 (n =212) | *p* |
| --- | --- | --- | --- |
| Tracheostomy care | 5 (50) | 2 (5) | 0.130 |
| Controlled ventilation with or without PEEP combined with IMV or assisted ventilation | 98 (1055) | 93 (197) | <0.001 |
| Cardiac arrest or countershock within 48 h | 91 (976) | 86 (182) | 0.023 |
| Controlled ventilation with intermittent or continuous muscle relaxants | 49 (530) | 28 (59) | <0.001 |
| Pulmonary artery catheter | 25 (271) | 22 (46) | 0.272 |
| Pacemaker on standby | 7 (78) | 14 (30) | 0.001 |
| Hemofiltration/dialytic techniques | 9 (101) | 9 (19) | 0.837 |
| Induced hypothermia | 42 (450) | 16 (34) | <0.001 |
| Intra-aortic balloon pressure | 5 (51) | 8 (16) | 0.094 |
| Emergency endoscopy or bronchoscopy | 23 (249) | 18 (39) | 0.125 |
| Vasoactive drug infusion (> 1 drug) | 47 (503) | 38 (80) | 0.015 |
| Intravenous alimentation | 27 (286) | 20 (43) | 0.052 |
| Frequent infusions of blood products (>5 U/24h) | 5 (54) | 7 (14) | 0.350 |
| Vasoactive drug infusion (1 drug) | 83 (891) | 81 (172) | 0.502 |
| Continuous antiarrhythmic infusions | 20 (212) | 17 (35) | 0.273 |
| Cardioversion for arrhythmia | 9 (94) | 8 (16) | 0.564 |
| Arterial line | 100 (1071) | 100 (211) | 0.432 |
| Measurement of cardiac output by any method | 36 (390) | 23 (48) | <0.001 |
| Active diuresis for fluid overload or cerebral edema | 61 (658) | 68 (143) | 0.092 |
| Active treatment for metabolic acidosis | 12 (127) | 15 (31) | 0.259 |
| Active anticoagulation (initial 48h) | 80 (858) | 71 (150) | 0.003 |
| Seizure treatment | 13 (140) | 6 (13) | 0.004 |
| Central venous pressure | 74 (795) | 58 (123) | <0.001 |
| Hemodialysis in unstable patient | 2 (18) | 4 (8) | 0.048 |
| Gastrointestinal feedings | 31 (337) | 25 (53) | 0.064 |
| ECG monitoring | 100 (1072) | 100 (212) | 0.657 |
| Hourly vital signs | 100 (1071) | 100 (212) | 0.529 |
| Chronic anticoagulation | 63 (679) | 54 (114) | 0.009 |
| Gastrointestinal decompression | 91 (975) | 80 (170) | <0.001 |
| PEEP = Positive end-expiratory pressure, IMV = Intermittent mandatory ventilation, ECG = electrocardiogram | | | |
