# Supplementary material for: Intensive care–treated cardiac arrest: a retrospective study on the impact of extended age on mortality, neurological outcome, received treatments and healthcare-associated costs
Source: Scand J Trauma Resusc Emerg Med. 2021 Jul 28;29:103. doi: 10.1186/s13049-021-00923-0 (PMC8317381; doi:10.1186/s13049-021-00923-0)
Supplement: Supplementary file 2 — Table of TISS-point distribution for individual procedures (OHCA-cases only). [file 13049_2021_923_MOESM2_ESM.docx]

**Additional file 2.** Table of TISS-point distribution for individual procedures (OHCA-cases only)

| Procedure, % (n) | Age <75 (n=691) | Age ≥75 (n=92) | *p* |
| --- | --- | --- | --- |
| Tracheostomy care | 3 (20) | 0 (0) | 0.098 |
| Controlled ventilation with or without PEEP combined with IMV or assisted ventilation | 100 (688) | 97 (89) | 0.003 |
| Cardiac arrest or countershock within 48 h | 92 (632) | 82 (75) | 0.002 |
| Controlled ventilation with intermittent or continuous muscle relaxants | 61 (421) | 37 (34) | <0.001 |
| Pulmonary artery catheter | 1 (5) | 0 (0) | 0.413 |
| Pacemaker on standby | 3 (21) | 8 (7) | 0.027 |
| Hemofiltration/dialytic techniques | 3 (23) | 0 (0) | 0.076 |
| Induced hypothermia | 62 (429) | 35 (32) | <0.001 |
| Intra-aortic balloon pressure | 2 (13) | 2 (2) | 0.847 |
| Emergency endoscopy or bronchoscopy | 19 (133) | 14 (13) | 0.236 |
| Vasoactive drug infusion (> 1 drug) | 46 (316) | 41 (38) | 0.423 |
| Intravenous alimentation | 24 (166) | 15 (14) | 0.059 |
| Frequent infusions of blood products (>5 U/24h) | 2 (15) | 1 (1) | 0.490 |
| Vasoactive drug infusion (1 drug) | 84 (579) | 84 (77) | 0.981 |
| Continuous antiarrhythmic infusions | 15 (100) | 12 (11) | 0.516 |
| Cardioversion for arrhythmia | 6 (43) | 5 (5) | 0.767 |
| Arterial line | 100 (690) | 99 (91) | 0.093 |
| Measurement of cardiac output by any method | 36 (249) | 21 (19) | 0.003 |
| Active diuresis for fluid overload or cerebral edema | 61 (418) | 74 (68) | 0.013 |
| Active treatment for metabolic acidosis | 7 (48) | 8 (7) | 0.815 |
| Active anticoagulation (initial 48h) | 87 (599) | 76 (70) | 0.007 |
| Treatment of seizures | 14 (96) | 11 (10) | 0.426 |
| Central venous pressure | 76 (528) | 57 (52) | <0.001 |
| Hemodialysis in unstable patient | 0 (1) | 0 (0) | 0.715 |
| Gastrointestinal feedings | 30 (204) | 28 (26) | 0.803 |
| ECG monitoring | 100 (691) | 100 (92) | - |
| Hourly vital signs | 100 (691) | 100 (92) | - |
| Chronic anticoagulation | 66 (453) | 54 (50) | 0.035 |
| Gastrointestinal decompression | 95 (657) | 92 (85) | 0.277 |
| PEEP = Positive end-expiratory pressure, IMV = Intermittent mandatory ventilation, ECG = electrocardiogram | | | |
