# Supplementary material for: Intensive care–treated cardiac arrest: a retrospective study on the impact of extended age on mortality, neurological outcome, received treatments and healthcare-associated costs
Source: Scand J Trauma Resusc Emerg Med. 2021 Jul 28;29:103. doi: 10.1186/s13049-021-00923-0 (PMC8317381; doi:10.1186/s13049-021-00923-0)
Supplement: Supplementary file 3 — Table of TISS-point distribution for individual procedures (IHCA-cases only). [file 13049_2021_923_MOESM3_ESM.docx]

**Additional file 3.** Table of TISS-point distribution for individual procedures (IHCA-cases only)

| Procedure, % (n) | Age <75 (n=382) | Age ≥75 (n=120) | *p* |
| --- | --- | --- | --- |
| Tracheostomy care | 8 (30) | 4 (5) | 0.167 |
| Controlled ventilation with or without PEEP combined with IMV or assisted ventilation | 96 (367) | 90 (108) | 0.010 |
| Cardiac arrest or countershock within 48 h | 90 (344) | 90 (107) | 0.779 |
| Controlled ventilation with intermittent or continuous muscle relaxants | 29 (109) | 21 (25) | 0.096 |
| Pulmonary artery catheter | 1 (5) | 0 (0) | 0.208 |
| Pacemaker on standby | 15 (57) | 19 (23) | 0.268 |
| Hemofiltration/dialytic techniques | 20 (78) | 16 (84) | 0.267 |
| Induced hypothermia | 6 (21) | 2 (2) | 0.080 |
| Intra-aortic balloon pressure | 10 (38) | 12 (14) | 0.590 |
| Emergency endoscopy or bronchoscopy | 30 (116) | 22 (26) | 0.065 |
| Vasoactive drug infusion (> 1 drug) | 49 (187) | 35 (42) | 0.007 |
| Intravenous alimentation | 31 (120) | 24 (29) | 0.130 |
| Frequent infusions of blood products (>5 U/24h) | 10 (39) | 11 (13) | 0.845 |
| Vasoactive drug infusion (1 drug) | 82 (312) | 80 (95) | 0.541 |
| Continuous antiarrhythmic infusions | 29 (112) | 20 (24) | 0.045 |
| Cardioversion for arrhythmia | 13 (51) | 9 (11) | 0.224 |
| Arterial line | 100 (381) | 100 (120) | 0.575 |
| Measurement of cardiac output by any method | 37 (141) | 24 (29) | 0.010 |
| Active diuresis for fluid overload or cerebral edema | 63 (240) | 63 (75) | 0.948 |
| Active treatment for metabolic acidosis | 21 (79) | 20 (24) | 0.872 |
| Active anticoagulation (initial 48h) | 68 (259) | 67 (80) | 0.817 |
| Treatment of seizures | 12 (44) | 3 (3) | 0.003 |
| Central venous pressure | 70 (267) | 59 (71) | 0.029 |
| Hemodialysis in unstable patient | 5 (17) | 7 (8) | 0.330 |
| Gastrointestinal feedings | 35 (133) | 23 (93) | 0.012 |
| ECG monitoring | 100 (381) | 100 (120) | 0.575 |
| Hourly vital signs | 100 (380) | 100 (120) | 0.427 |
| Chronic anticoagulation | 59 (226) | 53 (64) | 0.259 |
| Gastrointestinal decompression | 83 (318) | 71 (85) | 0.003 |
| PEEP = Positive end-expiratory pressure, IMV = Intermittent mandatory ventilation, ECG = electrocardiogram | | | |
