# Supplementary material for: Intensive care–treated cardiac arrest: a retrospective study on the impact of extended age on mortality, neurological outcome, received treatments and healthcare-associated costs
Source: Scand J Trauma Resusc Emerg Med. 2021 Jul 28;29:103. doi: 10.1186/s13049-021-00923-0 (PMC8317381; doi:10.1186/s13049-021-00923-0)

**Additional file 4.** Mean cost in euro based on initial rhythm

a) shockable rhythms


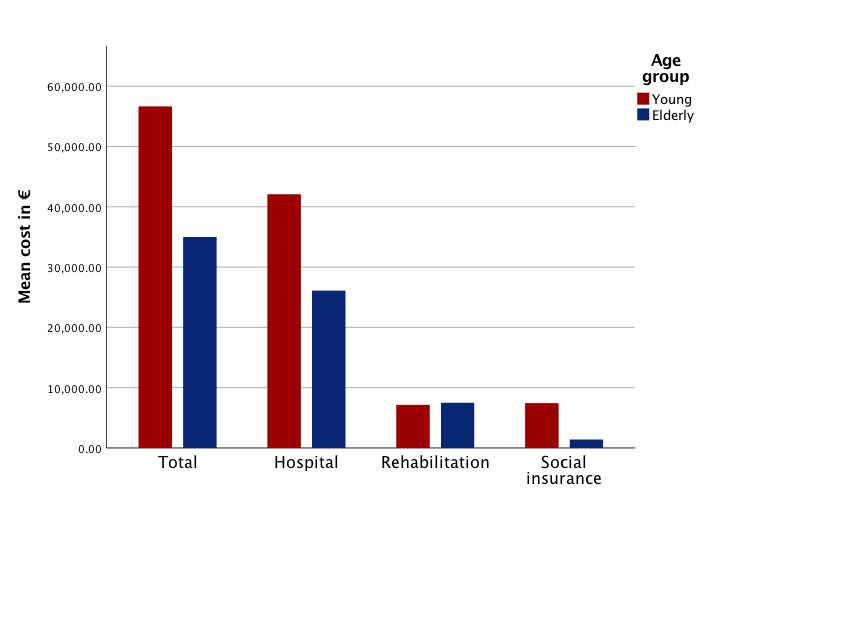


1. non-shockable rhythms
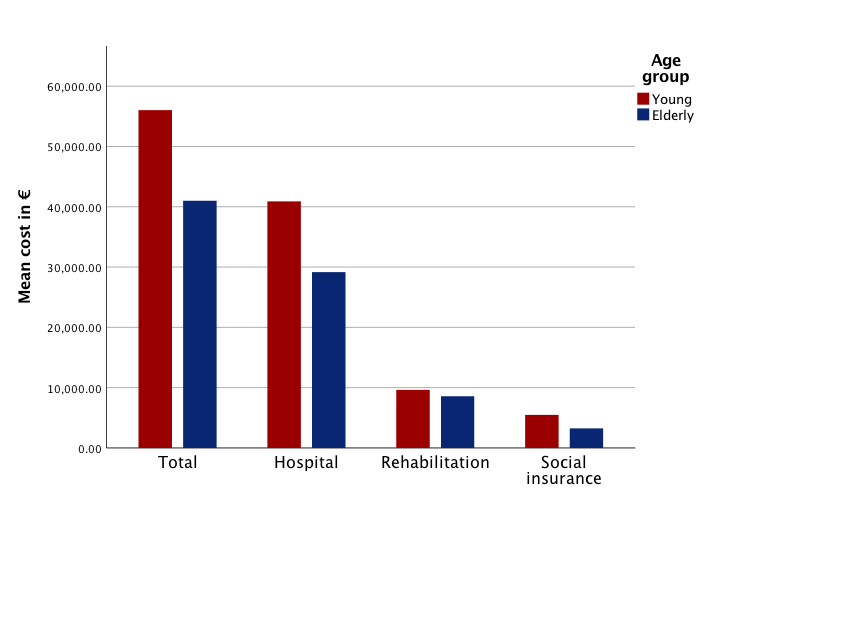

Supplement: Supplementary file 4 — Mean cost in euro based on initial rhythm (A) shockable rhythms (B) non-shockable rhythms. [file 13049_2021_923_MOESM4_ESM.docx]
