# Supplementary material for: Intensive care–treated cardiac arrest: a retrospective study on the impact of extended age on mortality, neurological outcome, received treatments and healthcare-associated costs
Source: Scand J Trauma Resusc Emerg Med. 2021 Jul 28;29:103. doi: 10.1186/s13049-021-00923-0 (PMC8317381; doi:10.1186/s13049-021-00923-0)
Supplement: Supplementary file 5 — Multivariate models for risk factors predicting (A) total costs (B) hospital costs (C) rehabilitation costs (D) Social Insurance Institution costs. [file 13049_2021_923_MOESM5_ESM.docx]

**Additional file 5.** Univariate models and multivariate models for risk factors predicting

a) total costs

|  | Multivariate model | |
| --- | --- | --- |
| Variable | OR (95% CI) | *p* |
| Age |  |  |
| Young (<75y) | 1 |  |
| Elderly (>=75) | -17 000 (-25 000- -7 800) | <0.001 |
| Woman | -5 400 (-13 000-1 700) | 0.137 |
| Initial CA-rhythm |  |  |
| Non-shockable | 1 |  |
| Shockable | 6 500 (-1 100-14 000) | 0.093 |
| Location of arrest |  |  |
| OHCA | 1 |  |
| IHCA | 13 000 (4 700-33 000) | 0.002 |
| ICUCA | 47 000 (33 000-60 000) | <0.001 |
| Time to ROSC in 10 minutes | -2 900 (-6 400-620) | 0.106 |
| APACHE II-score excluding age points^1^ | -4 900 (-9 000- -800) | 0.019 |

b) hospital costs

|  | Multivariate model | |
| --- | --- | --- |
| Variable | OR (95% CI) | *p* |
| Age |  |  |
| Young (<75y) | 1 |  |
| Elderly (>=75) | -12 000 (-18 000- -6 100) | <0.001 |
| Woman | -3 300 (-8 200-1 500) | 0.181 |
| Initial CA-rhythm |  |  |
| Non-shockable | 1 |  |
| Shockable | 7 000 (-1 900-12 000) | 0.008 |
| Location of arrest |  |  |
| OHCA | 1 |  |
| IHCA | 11 000 (5 500-17 000) | <0.001 |
| ICUCA | 36 000 (27 000-46 000) | <0.001 |
| Time to ROSC in 10 minutes | -2 000 (-4 400-430) | 0.107 |
| APACHE II-score excluding age points^1^ | -2 500 (-5 400-300) | 0.080 |

c) rehabilitation costs

|  | Multivariate model | |
| --- | --- | --- |
| Variable | OR (95% CI) | *p* |
| Age |  |  |
| Young (<75y) | 1 |  |
| Elderly (>=75) | -630 (-4 400- -3 200) | 0.744 |
| Woman | -1 300 (-4 400-1 800) | 0.414 |
| Initial CA-rhythm |  |  |
| Non-shockable | 1 |  |
| Shockable | 1 900 (-5 200-1 400) | 0.270 |
| Location of arrest |  |  |
| OHCA | 1 |  |
| IHCA | 1 000 (-2 600- 4 700) | 0.584 |
| ICUCA | 8 600 (2 800-1 400) | 0.004 |
| Time to ROSC in 10 minutes | -730 (-2 300-790) | 0.346 |
| APACHE II-score excluding age points^1^ | -1 600 (-3 400-250) | 0.091 |

d) Social Insurance Institution costs

|  | Multivariate model | |
| --- | --- | --- |
| Variable | OR (95% CI) | *p* |
| Age |  |  |
| Young (<75y) | 1 |  |
| Elderly (>=75) | -3 800 (-5 500- -2 100) | <0.001 |
| Woman | -770 (-2 200-640) | 0.284 |
| Initial CA-rhythm |  |  |
| Non-shockable | 1 |  |
| Shockable | 1 300 (-180-2 800) | 0.085 |
| Location of arrest |  |  |
| OHCA | 1 |  |
| IHCA | 830 (-820-2 500) | 0.326 |
| ICUCA | 1 600 (-1 000-4 200) | 0.229 |
| Time to ROSC in 10 minutes | -180 (-870-510) | 0.615 |
| APACHE II-score excluding age points^1^ | -850 (-1 700- -30) | 0.080 |
