# Supplementary material for: Intensive care–treated cardiac arrest: a retrospective study on the impact of extended age on mortality, neurological outcome, received treatments and healthcare-associated costs
Source: Scand J Trauma Resusc Emerg Med. 2021 Jul 28;29:103. doi: 10.1186/s13049-021-00923-0 (PMC8317381; doi:10.1186/s13049-021-00923-0)
Supplement: Supplementary file 6 — KM-curves based on location of arrest (A) all cases during the whole follow up-period, Log Rank p < 0.001 (B) OHCA, Log Rank p < 0.001 (C) IHCA, Log Rank p = 0.003 (D) ICUCA, Log Rank p = 0.079. [file 13049_2021_923_MOESM6_ESM.docx]

**Additional file 5.** KM-curves based on location of arrest

1. All cases during the whole follow up-period, Log Rank *p* < 0.001

**
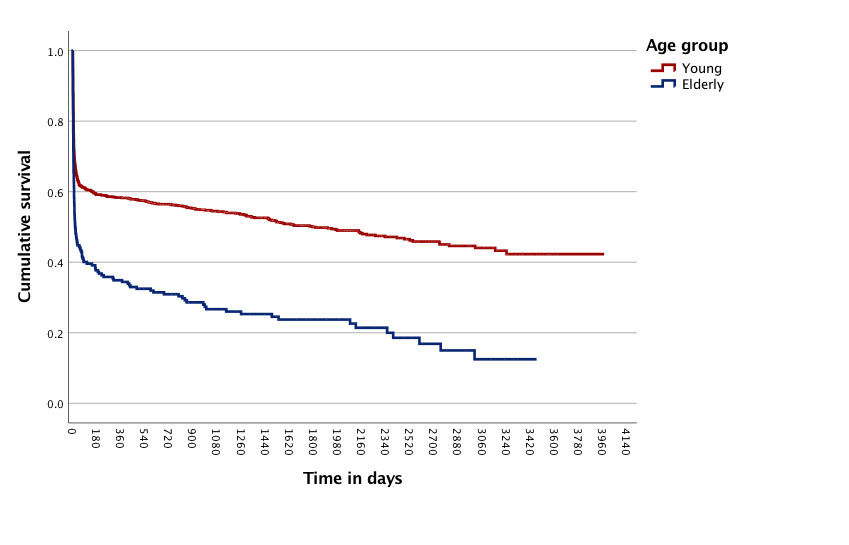
**

1. OHCA, Log Rank *p* < 0.001
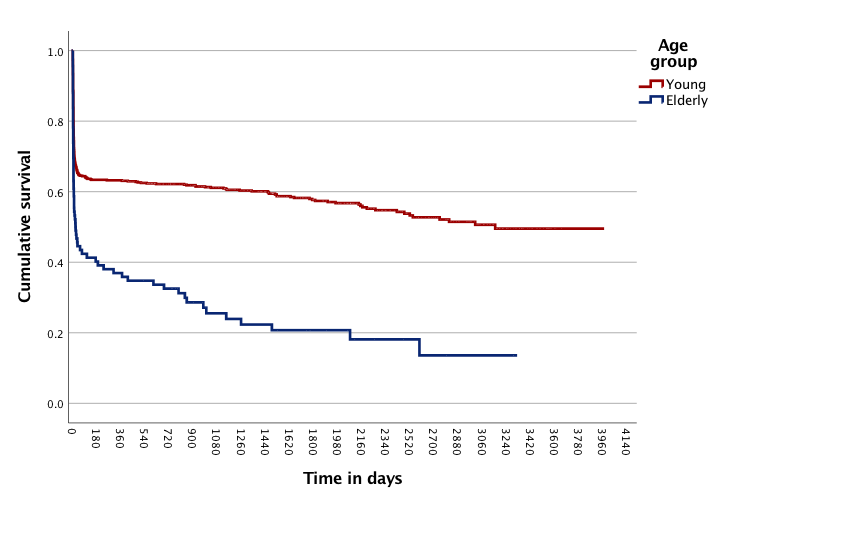

2. IHCA, Log Rank *p* = 0.003


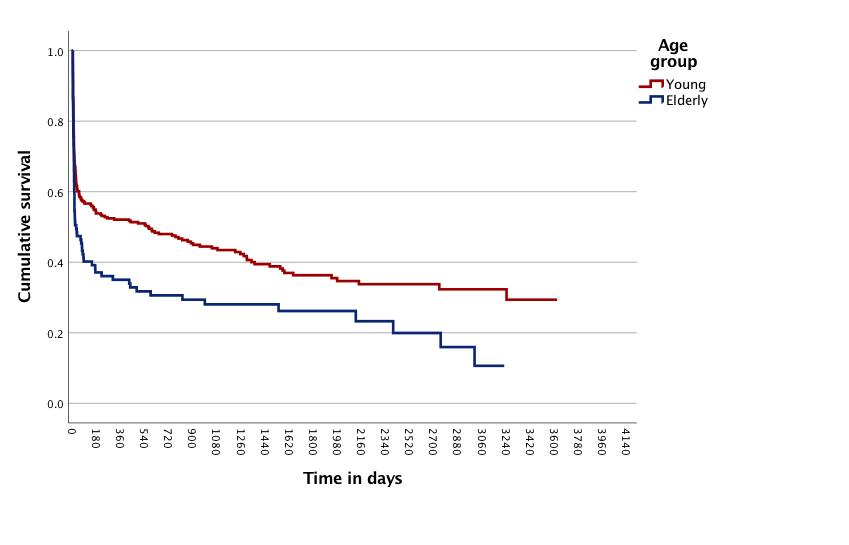


1. ICUCA, Log Rank *p* = 0.079

**
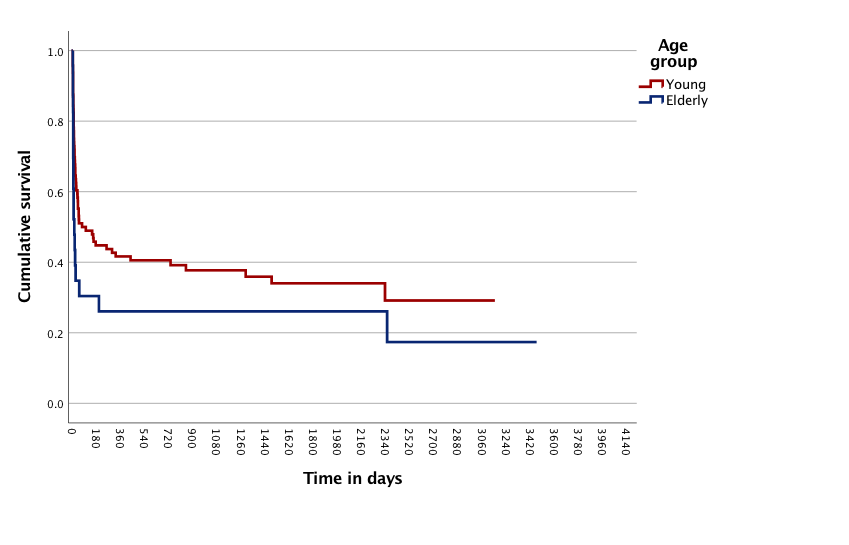
**
