# Supplementary material for: Intensive care–treated cardiac arrest: a retrospective study on the impact of extended age on mortality, neurological outcome, received treatments and healthcare-associated costs
Source: Scand J Trauma Resusc Emerg Med. 2021 Jul 28;29:103. doi: 10.1186/s13049-021-00923-0 (PMC8317381; doi:10.1186/s13049-021-00923-0)

**Additional file 6.** KM-curves based on initial rhythm

1. Shockable rhythm (VF/VT), Log Rank *p* < 0.001
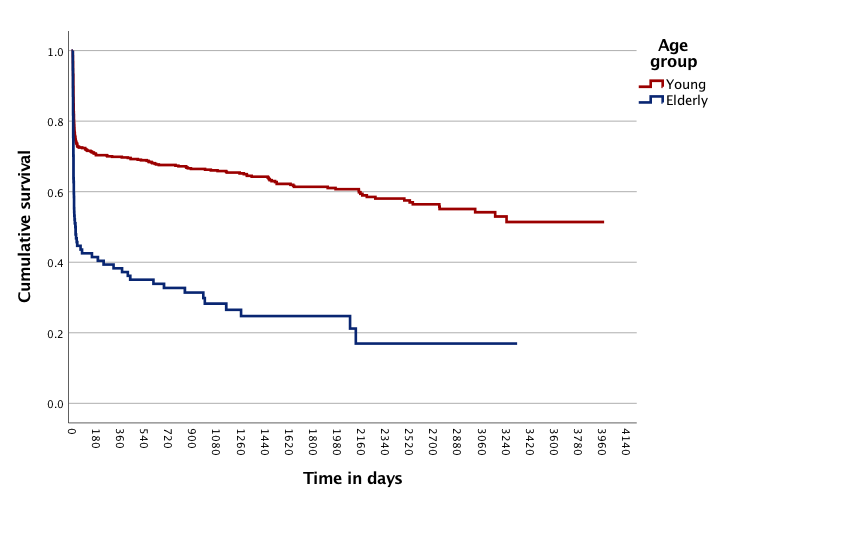

2. Non-shockable rhythm, Log Rank *p* = 0.062


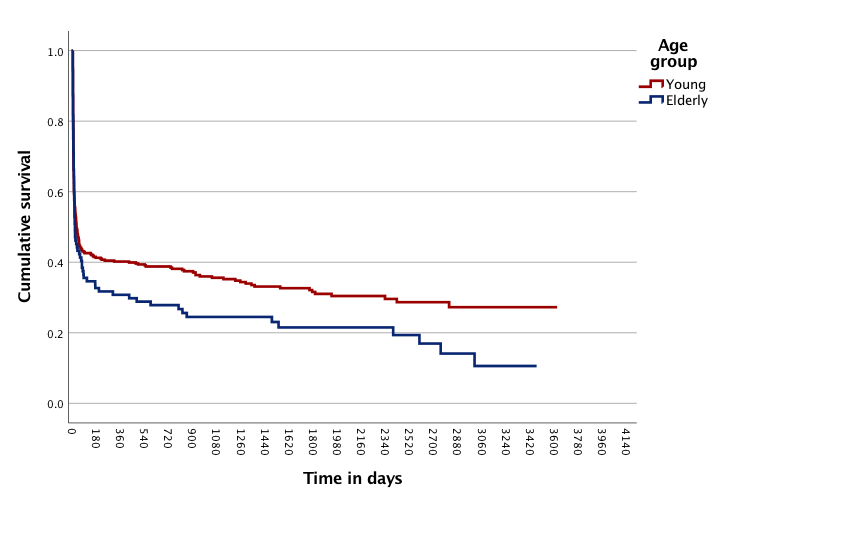

Supplement: Supplementary file 7 — KM-curves based on initial rhythm (A) Shockable rhythm (VF/VT), Log rank p < 0.001 (B) Non-shockable rhythm, Log Rank p = 0.062. [file 13049_2021_923_MOESM7_ESM.docx]
